# Synaptic synergies and their role in integrating distinct synaptic pathways

**DOI:** 10.1186/1471-2202-14-S1-P140

**Published:** 2013-07-08

**Authors:** Willem AM Wybo, Benjamin Torben-Nielsen, Marc-Oliver Gewaltig

**Affiliations:** 1 Blue Brain Project, Brain Mind Institute, EPFL, Lausanne, Switzerland

## 

Faithful reconstructions of cortical microcircuits allow the characterization of cortical synaptic pathways along with the specific dendritic regions that they target on the post-synaptic neurons [2]. But given this information, the question remains how neurons integrate or discriminate between distinct inputs impinging at spatially segregated locations on their dendrites. Here, we investigated how and under which circumstances synaptic pathways targeting distinct regions in the dendrites can interact.

We followed an analytical approach, starting with a Volterra expansion of the generalized cable equation. In this expansion, the second order kernel is the lowest-order kernel that is sensitive to correlations between spatially segregated input locations. It, thus, provides insight in the spatial extent of local dendritic computation. Our approach is similar to that of Kistler and Gerstner [3]. For fairly general dendritic geometries and distributions of HH-type ion channels, we show that the second-order Volterra kernel can be computed from the first-order kernel, known for linear systems as the Green's function [1,4]. We implemented and tested our analytical results, using morphologically detailed neuron models. Thus, we have obtained a tool for analyzing distance scales of spatial correlations in detailed neuron models.

Using our method, we can identify input regions that collaborate (correlate) to produce an output. Using the recently described synaptic pathways, we elaborate on the dendritic regions where interaction can occur and show how these interactions are shaped by ion channel distribution and dendritic geometry. Finally we discuss the advantages and limitations of our method.
